# Survival marker genes of colorectal cancer derived from consistent transcriptomic profiling

**DOI:** 10.1186/s12864-018-5193-9

**Published:** 2018-12-11

**Authors:** Jorge Martinez-Romero, Santiago Bueno-Fortes, Manuel Martín-Merino, Ana Ramirez de Molina, Javier De Las Rivas

**Affiliations:** 1 Bioinformatics and Functional Genomics Group, Cancer Research Center (CiC-IBMCC, CSIC/USAL/IBSAL), Consejo Superior de Investigaciones Cientificas (CSIC) and University of Salamanca (USAL), Salamanca, Spain; 2 Molecular Oncology and Nutritional Genomics of Cancer Group, Precision Nutrition and Cancer Program, IMDEA Food Institute (CEI, UAM/CSIC), Madrid, Spain; 3 Department of Computer Science, Universidad Pontificia de Salamanca (UPSA), Salamanca, Spain

**Keywords:** Cancer, Colorectal cancer, Colon, Survival, Kaplan-Meier analysis, Gene marker, Bioinformatics, Transcriptomics, Gene Expression

## Abstract

**Background:**

Identification of biomarkers associated with the prognosis of different cancer subtypes is critical to achieve better therapeutic assistance. In colorectal cancer (CRC) the discovery of stable and consistent survival markers remains a challenge due to the high heterogeneity of this class of tumors. In this work, we identified a new set of gene markers for CRC associated to prognosis and risk using a large unified cohort of patients with transcriptomic profiles and survival information.

**Results:**

We built an integrated dataset with 1273 human colorectal samples, which provides a homogeneous robust framework to analyse genome-wide expression and survival data. Using this dataset we identified two sets of genes that are candidate prognostic markers for CRC in stages III and IV, showing either up-regulation correlated with poor prognosis or up-regulation correlated with good prognosis. The top 10 up-regulated genes found as survival markers of poor prognosis (i.e. low survival) were: DCBLD2, PTPN14, LAMP5, TM4SF1, NPR3, LEMD1, LCA5, CSGALNACT2, SLC2A3 and GADD45B. The stability and robustness of the gene survival markers was assessed by cross-validation, and the best-ranked genes were also validated with two external independent cohorts: one of microarrays with 482 samples; another of RNA-seq with 269 samples. Up-regulation of the top genes was also proved in a comparison with normal colorectal tissue samples. Finally, the set of top 100 genes that showed overexpression correlated with low survival was used to build a CRC risk predictor applying a multivariate Cox proportional hazards regression analysis. This risk predictor yielded an optimal separation of the individual patients of the cohort according to their survival, with a *p*-value of 8.25e-14 and Hazard Ratio 2.14 (95% CI: 1.75–2.61).

**Conclusions:**

The results presented in this work provide a solid rationale for the prognostic utility of a new set of genes in CRC, demonstrating their potential to predict colorectal tumor progression and evolution towards poor survival stages. Our study does not provide a fixed gene signature for prognosis and risk prediction, but instead proposes a robust set of genes ranked according to their predictive power that can be selected for additional tests with other CRC clinical cohorts.

**Electronic supplementary material:**

The online version of this article (10.1186/s12864-018-5193-9) contains supplementary material, which is available to authorized users.

## Background

Colorectal cancer (CRC) is one of the most frequent tumors that causes great morbidity worldwide. It is the third most common cancer in men, the second most common cancer in women and the third leading cause of global cancer mortality (https://www.wcrf.org/). CRC is a heterogeneous disease since from one patient to another it differs in clinical presentation, molecular characteristics, and prognosis [1]. The heterogeneity of CRC increases the complexity of this tumoral pathology, making subtyping and stratification a difficult task for therapeutic decisions. In this way, personalized medicine for CRC is becoming increasingly needed, especially for targeted therapies where large variations between individual’s treatment responses exist [1, 2]. In this context, the need to find robust gene markers associated with specific subtypes of CRC led us to this study. Furthermore, the specific purpose of our work was to find consistent biomolecular targets that, together to facilitate samples stratification, could be related to the prognosis of the disease using survival data.

The genomic and transcriptomic profiling of human cancer samples has been demonstrated over the last decade as an excellent way to obtain a better molecular characterization of many tumor types and subtypes. While gene expression-based CRC classifications has been heavily approached [2], little consensus in CRC standalone gene bio-marking has been achieved. In fact, several studies have identified a broad variety of gene sets as gene expression profiles for classification and categorization of this malignant disorder [3, 4]. Moreover, several transcriptomic-based tests oriented towards prognosis have also been investigated. Some examples of these are: *ColoLipidGene* [5], *ColoGuidePro* [6] or *ColoPrint* [7]; that include gene signatures associated with CRC survival in some specific biological contexts. Despite these efforts, at present there is not a clear compendium of gene markers for CRC survival and it is quite difficult to find consistency in the literature.

In the clinic, patients are classified into four CRC stages based in the anatomo-pathologycal characteristics of their tumors. It is common to use the *TNM Staging System* (where **T** stands for tumor, **N** for lymph node, and **M** for metastasis). The disease “staging” also allows grouping the patients in 4 progressive cancer stages, indicated by roman numerals: **I**, **II**, **III**, and **IV** [8]. In this way, stages I and II correspond to cases which had not shown cancer cells beyond the tumor or blood. By contrast, stages III and IV correspond to individuals in where the cancer had diseminate to the lymph system or other organs in the body. This four stage categorization represents significantly distinctive patients groups for final outcome or disease relapse, but the stages do not predict the risk of each individual patient because they are not directly associated to survival [9].

Based on the described need and potential benefits to find survival marker genes correlated with high risk and poor prognosis in CRC; we investigated global gene expression profiles of colorectal tumors and its alteration throughout stages, to identify genes that could be levered as biomarkers of survival and prognosis for CRC in late stages (i.e., III and IV). To undertake this work we performed a deep analysis on a large cohort of human samples derived from a robust integration of several datasets that had transcriptomic and clinical survival data. The integration provided a homogeneous and well-standardized meta-dataset that includes 1273 human colorectal samples. The identification of candidate markers was performed using an initial contrast between the gene expression of the subset of patients with CRC allocated by their clinical features to stages I and II versus the patients with tumors corresponding to stages III and IV. Finally, after internal and external cross-validation, the genes selected as best survival markers were used to construct a risk predictor to allow stratification of the patients with respect to their relative risk.

## Results

### A large dataset of CRC samples including global expression and survival data

We first built a large cohort of CRC samples collected from individuals that had clinical record with survival data times, as well as genome-wide expression profiles of their colorectal primary tumors at diagnosis (i.e. before any drug treatment). Our aim was to achieve a meta-dataset with at least 1 thousand samples and to demonstrate a good integration of the global transcriptomic profiles of different samples sets avoiding the typical batch-effects that can alterate any unified analysis.

Table 1 presents the datasets of CRC samples that were collected to produce the integrated dataset analysed in this work. All the CRC samples included in this meta-dataset were tested for global gene expression profiling using the platform of high-density microarrays from *Affymetrix*: Human Genome U133 Plus 2.0. Using this platform, the probesets of the arrays were mapped to single genes (as indicated in Risueño et al.) [10] and, in this way, each microarray measured the expression signal of 20,079 human genes (using the mapping provided by the Chip Description File, CDF v.21 from: http://brainarray.mbni.med.umich.edu/Brainarray/Database/CustomCDF/).

**Table 1:** Summary information about the series of colorectal cancer (CRC) samples that were collected to produce the integrated data set analyzed in this work

GEO dataset	Sample Source	Sample Description	Total samples in dataset	PubMed PMID	Authors and Year	Samples discarded	Samples processed
GSE14333	Royal Melbourne Hospital, Western Hospital and Peter MacCallum Cancer Center, AUSTRALIA. H Lee Moffitt Cancer Center, USA	primary colorectal cancers	290	19996206	Jorissen RN et al. (2009)	64	226
GSE17536	Moffitt Cancer Center, USA	colorectal cancer patients	177	19914252	Smith JJ et al. (2010)	0	177
GSE31595	Roskilde Hospital, DENMARK	patients with stage II and III colorectal cancer	37	–	Thorsteinsson M et al. (2011)	0	37
GSE33113	Academic Medical Center in Amsterdam, NETHERLANDS	primary tumor resections from stage II colorectal patients	90	22496204	Kemper K et al. (2012)	0	90
GSE38832	Vandervilt University Medical Center, USA	tumor samples collected from colorectal patients	122	25320007	Tripathi MK et al. (2014)	0	122
GSE39084	Toulouse Hospital, FRANCE	sporadic early onset primary colorectal carcinomas	70	25083765	Kirzin S et al. (2014)	1	69
GSE39582	Institut G. Roussy (Villejuif), Hosp. Saint Antoine (Paris), Hosp. G.Pompidou (Paris), Hosp. Hautepierre (Strasbourg), Hosp. Purpan (Toulouse), Institut P. Calmettes (Marseille), Centre Antoine Lacassagne (Nice), FRANCE	colorectal cancer samples	566	23700391	Marisa L et al. (2013)	14	552
Total number			1352				1273

All the CRC samples were tested for global gene expression profiling using high-density microarrays Human Genome U133 Plus 2.0 from *Affymetrix* (that measure the signal of 20,141 human genes). The total collection included 1352 samples, but only 1273 were finally used. A group of 79 samples were discarded because they did not have survival data or they presented anomalous data distributions with respect to the other samples of the same series

As a whole, Table 1 includes 7 series that were obtained from the Gene Expression Omnibus repository (GEO, https://www.ncbi.nlm.nih.gov/geo/). These datasets included a total amount of 1352 CRC samples, but after collecting the clinical survival data and carrying out the integration and normalization protocols we finished with 1273 samples, since we filtered 79 samples that did not have survival data or did not show comparable data distributions after normalization. The phenotypic and clinical information about the final collection of 1273 samples, i.e., the available data about age, gender, survival time, location of the tumor, degree and TNM staging, presence of mutation in some cancer genes (TP53, KRAS, BRAF), etc.; is included in Additional file 1: Table S1. When information was not available for a given sample the table includes *not assigned* values (NA).

### Evaluation of normalization procedures to integrate independent batches

We performed the integration and combined normalization of the CRC expression datasets using 5 different procedures. The procedures applied different normalization algorithms to provide a homogeneous signal matrix, avoiding bias due to batch effect on the global expression profile of the CRC samples. The procedures applied were: **(i)** Robust Multi-array Average (RMA) algorithm [11]; **(ii)** RMA plus Combatting Batch effects (ComBat) algorithm [12]; **(iii)** Frozen Robust Multi-array Average (fRMA) algorithm [13]; **(iv)** fRMA plus Combat; **(v)** fRMA plus scaling of the data using mean-centered expression values.

**Fig. 1:**
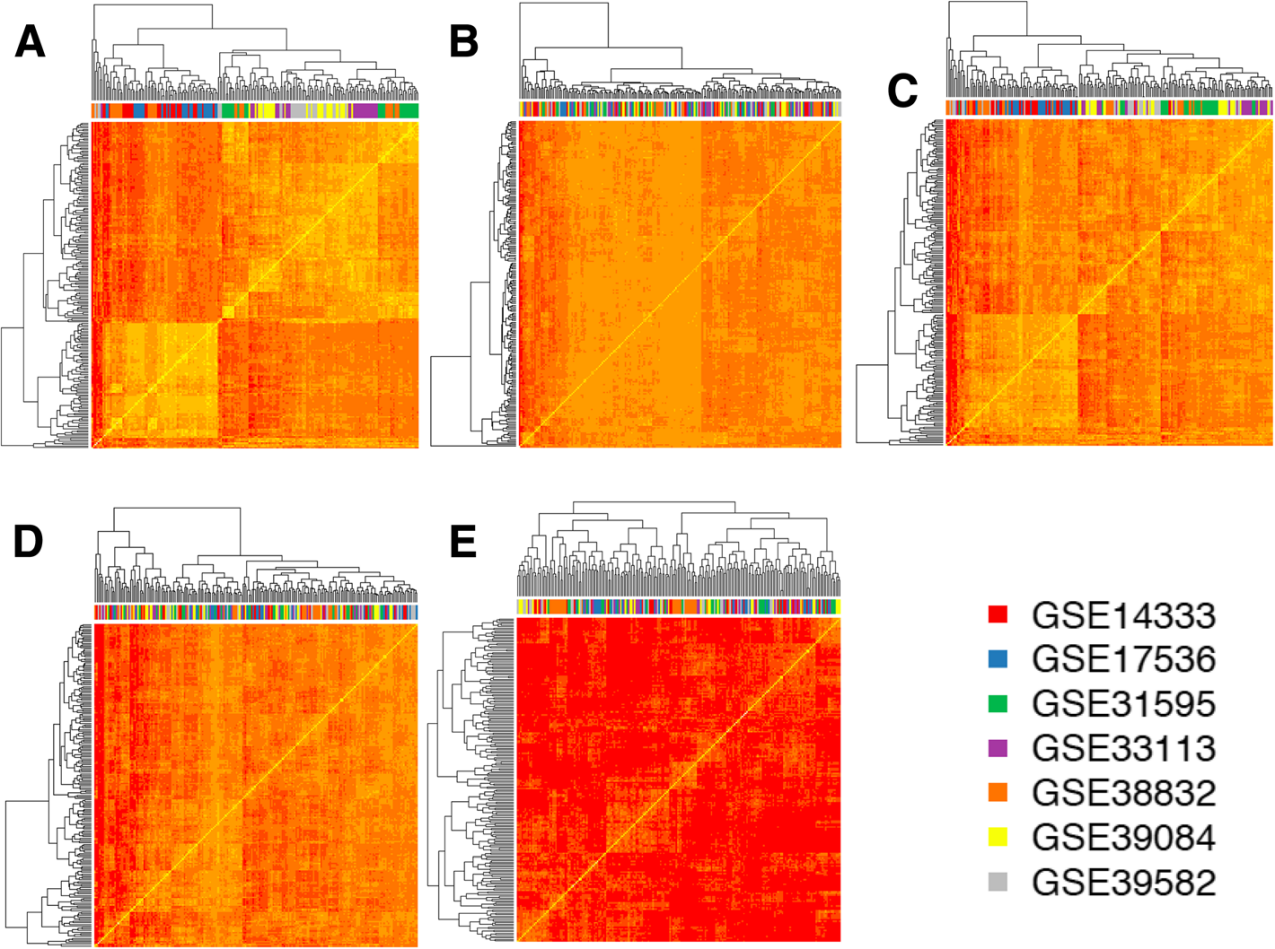
Symmetric heatmaps representing the similarity between the overall gene expression signal of the samples compared with each other. Each heatmap is composed of 210 samples (30 × 7, 30 samples random selected from each batch, i.e. from each one of the 7 GSE datasets). The samples of each batch are identified by a color in the top bar below the top dendrograms (following the colors legend). Each heatmap represents a different preprocessing and normalization method performed to merge the datasets in one batch. The methods applied were: **a** RMA; **b** RMA plus ComBat; **c** fRMA; **d** fRMA plus ComBat; **e** fRMA plus scaling of the data using mean-centered expression values

To evaluate and compare the results provided by each one of these 5 procedures we carried out several analyses. Figure 1 presents the heatmaps derived from an unsupervised clustering of the samples using in each case the expression data matrix derived from each one of the 5 procedures applied. Due to the fact that each series has a different number of samples (one with more than 500 and several other with less than 100), we did a random selection of an even number of samples for each dataset to be included in the cluster analysis: 30 samples from each one. In this way, each heatmap is composed of 210 samples (30 × 7): 30 samples from each one of the 7 datasets (identified by the ID number, GSE, from GEO). In Fig. 1 the samples of each batch are identified by a color that is indicated in the horizontal bar below the dendrograms. Each heatmap represents a different preprocessing and normalization method performed to merge the datasets in one meta-dataset. The results shown in these clustering analyses indicate that in the case of methods that gave the heatmaps A, C and E, several samples of the same color are grouped together showing that they have a common correlation profile within the global expression signature. By contrast, in the case of methods that gave the heatmaps B and D, there is a clearer shuffling of all the colors, which reflects a homogenous mix of the overall expression signal coming from different datasets.

**Fig. 2:**
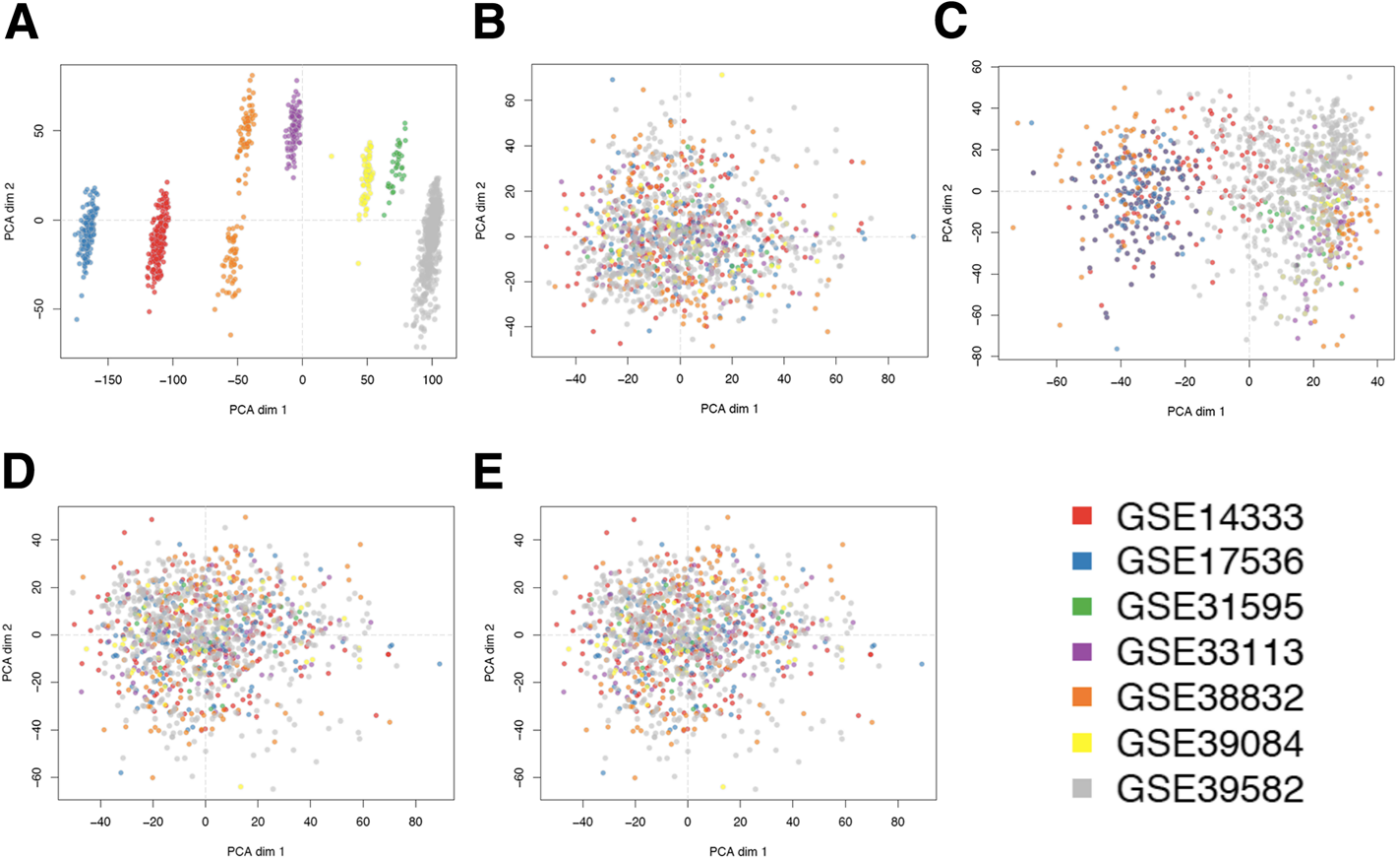
Plots presenting the distribution of the 1273 samples from 7 datasets (GSEs) obtained by Principal Component Analysis (PCA) of the global gene expression profile of each sample; that converts the signal of each sample using an orthogonal transformation in linearly uncorrelated variables called principal components or dimensions. Each plot presents the values of the two main dimensions (dim 1 versus dim 2) and corresponds to the PCA results obtained using the expression data calculated with different preprocessing and normalization methods. The methods applied were: **a** RMA; **b** RMA plus ComBat; **c** fRMA; **d** fRMA plus ComBat; **e** fRMA plus scaling of the data using mean-centered expression values. The samples of each batch are identified by color dots following the colors legend

The clustering analysis presented in the symmetric heatmaps of Fig. 1 was done using, for each sample, a vector including the expression signals along all genes and calculating with these vectors the pair-wise *Pearson* correlations between samples and the pair-wise distance matrix derived from such correlations. This approach can reveal major effects associated to the global expression signal of the samples, but it is not very sensitive to detect minor changes in a small number of genes. For this reason we applied a second approach to compare the results provided by the 5 normalization procedures in order to select the one that produces the best unification of the 7 CRC datasets, preserving a good signal to noise ratio in the expression distributions. Algorithms of dimensionality reduction, such as PCA (Principal Component Analysis), allow exploring large datasets in an accurate way to identify factors that are relevant for the variance of studied variables (in our case the expression of the genes in the unified meta-dataset of 1273 samples). Figure 2 presents the plots derived from the PCA done over the 5 expression matrices (i.e. the signal of 20,079 genes in 1273 samples) obtained with 5 different normalization approaches. These results show very clearly that the RMA method (Fig. 2a) is not good to provide a proper normalization of different batches, since the samples keep a very strong signal associated to each batch. The fRMA method (Fig. 2c) neither is good, since some samples (specially the ones from the largest batch GSE39582) still keep a strong signal associated to their batch. By contrast, the analysis of the data provided by the other 3 procedures (RMA plus Combat, fRMA plus Combat and fRMA plus mean-centered scaling, Fig. 2b, d and e, respectively) showed an adequate mix of all the samples from different batches. Within these 3 procedures, the normalization is very similar keeping a good signal to noise ratio along the genes and a small signal reduction. We finally select option B, RMA plus Combat, because the heatmap in Fig. 1b showed the best mix between series and a better similarity between the samples (compared to options D or E).

As a final testing to identify the best integration and normalization procedure of the 7 CRC expression datasets, we carried out a linear regression analyses on the global expression matrix considering as predictors 7 independent dummy variables or factors. These variables correspond to the series from which each sample comes from. In this way, if these factors have a significant influence in the expression signal distributions, the linear regression analysis will show a significant *p*-value and correlation. The results of this analysis are presented in Table 2, that reveals again that only the data matrices produced by the methods B and D (RMA plus Combat and fRMA plus Combat, respectively) do not show a significant effect attributed to belonging to one of the series. Finally, we choose B versus D as the final procedure applied because, despite being very similar, the application of RMA plus Combat provoked less dramatic changes with respect to the raw signal expression.

**Table 2:** Results of the linear regression analyses on the global expression matrix calculated for the 1273 samples from 7 datasets (GSEs) combined using 5 different preprocessing and normalization methods

FACTORS considered	Estimated coefficients	std. error	t value	*p*.value	Factor effect
(A) RMA					
Intercept	6.925	0.014	512.610	<2e-16	–
(GSE14333+) GSE17536	0.387	0.019	20.230	<2e-16	yes
GSE31595	−1.212	0.019	−63.440	<2e-16	yes
GSE33113	−0.577	0.019	−30.210	<2e-16	yes
GSE38832	−0.355	0.019	−18.570	<2e-16	yes
GSE39084	−0.978	0.019	−51.180	<2e-16	yes
GSE39582	−1.375	0.019	−71.970	<2e-16	yes
(B) RMA plus Combat					
Intercept	6.219	0.013	473.582	<2e-16	–
(GSE14333+) GSE17536	0.000	0.019	0.001	0.999	no
GSE31595	0.002	0.019	0.122	0.903	no
GSE33113	0.001	0.019	0.051	0.959	no
GSE38832	−0.001	0.019	−0.033	0.973	no
GSE39084	0.002	0.019	0.092	0.927	no
GSE39582	0.001	0.019	0.029	0.977	no
(C) fRMA					
Intercept	6.535	0.015	450.434	<2e-16	–
(GSE14333+) GSE17536	−0.011	0.021	−0.553	0.580	no so much
GSE31595	0.089	0.021	4.329	0.000	yes
GSE33113	0.071	0.021	3.455	0.001	yes
GSE38832	0.054	0.021	2.641	0.008	yes
GSE39084	0.096	0.021	4.695	0.000	yes
GSE39582	0.089	0.021	4.336	0.000	yes
(D) fRMA plus Combat					
Intercept	6.590	0.014	457.338	<2e-16	–
(GSE14333+) GSE17536	0.000	0.020	0.001	1.000	no
GSE31595	0.002	0.020	0.093	0.926	no
GSE33113	0.001	0.020	0.072	0.942	no
GSE38832	0.000	0.020	0.019	0.985	no
GSE39084	0.002	0.020	0.089	0.929	no
GSE39582	0.000	0.020	0.007	0.994	no
(E) fRMA plus mean centered					
Intercept	0.000	0.000	−1.638	0.101	–
(GSE14333+) GSE17536	0.000	0.000	1.264	0.206	yes
GSE31595	0.000	0.000	0.288	0.773	no so much
GSE33113	0.000	0.000	1.605	0.108	yes
GSE38832	0.000	0.000	1.449	0.147	yes
GSE39084	0.000	0.000	−0.076	0.940	no
GSE39582	0.000	0.000	1.395	0.163	yes

The methods applied were: **(A)** RMA; **(B)** RMA plus ComBat; **(C)** fRMA; **(D)** fRMA plus ComBat; **(E)** fRMA plus scaling of the data using mean-centered expression values. The linear regression is done to evaluate the “batch effect” (i.e. considering that the tested factors are the fact of “belonging” to a given dataset). Thus, when the *p*-value of the factors are significant (< 0.05), the “batch effect” remains on the overall expression signal. A marginal low significance was considered when *p*-values were < 0.20 in the case E

### Identification of genes associated to advanced CRC that mark survival differences

Once we produced a large and well-integrated meta-dataset of CRC samples, having global expression profiles and clinical survival data for all cases, we proceed to the identification of the subset of genes that suffer significant changes with colorectal tumor progression. To do this, we explored the overall expression matrix to detect the genes that showed a significant expression change when comparing CRC tumors in early stages (stages I and II) versus CRC tumors in late or advanced stages (stages III and IV). This comparison was done applying LIMMA, differential expression algorithm, and retrieving all genes that gave a significant *p*-value (adjusted *p* < 0.05) in either direction (i.e., genes up-regulated with the progression of the disease, in late versus early CRC stages; or genes down-regulated with the progression of the disease). Such differential expression analysis gave a subset of 2707 human genes: 2524 corresponding to protein-coding genes and the rest to non-coding genes (in this work we focused only in the protein-coding genes).

Once we had the subset of genes that can be associated to advanced or progression of CRC, we perform a second analysis on these gene candidates to find out which ones can be correlated with the survival of the corresponding patient samples based on their expression signals. To do this, we carried out Kaplan-Meier (KM) analysis of the survival times of the set of 1273 colorectal cancer samples for each one of the 2524 genes found in the previous exploration. In this analysis, the genes were ranked considering the non-parametric log-rank test that evaluates the separation between the two KM curves for two prognostic groups: one with good survival and another with poor survival. To do this, our algorithm performs for each gene multiple splits of the sample cohort in two groups, and looks for the splitting that provides the best separation between groups (i.e. the best *p*-value). Then, a stringent cut-off value (adjusted *p* < 0.0003) was used to select the genes that are considered significant. This allowed the identification of 429 significant genes in which the overexpression correlated with low survival, plus 336 significant genes where the repression correlated with low survival. These analyses were done in a univariate mode, considering each gene as an independent factor.

**Fig. 3:**
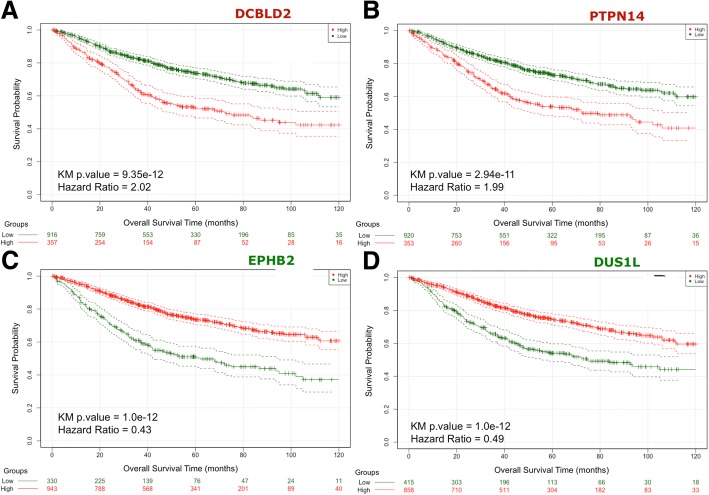
Kaplan-Meier plots of the survival analysis of the set of 1273 samples from colorectal cancer (CRC) patients. The patients are separated in two groups (high in red and low in green) according to the expression profiles of 4 genes: **a** DCBLD2, **b** PTPN14, **c** EPHB2, **d** DUS1L. These genes provided the best split between patients of high and low risk based in their expression levels. In the case of genes DCBLD2 and PTPN14 (labelled in red) the over-expression is correlated with poor survival; and in the case of genes EPHB2 and DUS1L (labelled in green) the over-expression is correlated with good survival. In all cases the adjusted p.values of the analyses are very significant (as indicated inside each plot), indicating that the two populations represented by the two curves have a very clear difference in their overall survival

Figure 3 shows the Kaplan-Meier plots corresponding to the survival profiles of the two populations of individuals that were segregated according to the expression values of the gene tested. The 4 plots correspond to the top genes: DCBLD2 and PTPN14 with overexpression correlated to low survival; and EPHB2 and DUS1L with repression correlated to low survival. The separation of the two populations in both cases is very significant, with KM *p*-values < 1.0e-10 and Hazard Ratios (HR) around 2.0 for overexpression cases and around 0.45 for repression cases. These parameters were calculated using all the 1273 samples; however it was necessary to do an internal cross-validation of these results to assess how stable and reliable was the signal for each one of the selected genes.

We carried out a cross-validation of the top-200 genes selected in any of the two conditions (i.e. selected as survival markers when they were up-regulated for the cases of poor survival or when they were up-regulated for the cases of better survival). This internal cross-validation was done using for each gene a resampling strategy that randomly selected 80% of the sample 100 times (i.e. doing 100 iterations). The results corresponding to the top 100 genes are included in Additional file 2**:** Table S2, for the case of up-regulation for poor survival, and the other top 100 genes in Additional file 3: Table S3, for the case up-regulation for better survival.

A short view of these data is shown in Table 3 that presents the 50 genes selected as best survival markers of CRC: the first part of the table corresponds to the top 25 genes, where up-regulation corresponds to shorter survival and higher risk (HR > 1); the second part of the table corresponds to the top 25 genes, where up-regulation corresponds to longer survival and lower risk (HR < 1). The genes were ranked by their KM *p*-values and the HR values calculated for the whole dataset (i.e. for all the 1273 samples, all-dt). As indicated, the stability and robustness of the gene survival markers was assessed via a resampling strategy with random selection of 80% of the dataset 100 times. For the final ranking of the genes included in these tables we also considered that they had to give a significant adjusted p-value in more than 80 out of 100 bootstrap iterations (i.e. N-sinf-in-100i > 80).

**Table 3:** Genes selected as top-50 best *survival markers* of colorectal cancer (CRC)

Number	GENE ENSEMBL_ID	GENE Symbol	KM.*p*.value (all-dt)	HR (all-dt)	N-signf-in-100i (KM.p.value)	HR (mean-in-100i)	GENE HGNC_ID	GENE DESCRIPTION
1	ENSG00000057019	DCBLD2	0.0000000000	2.02	99	2.106	24627	discoidin; CUB and LCCL domain containing 2 [HGNC:24627]
2	ENSG00000152104	PTPN14	0.0000000000	1.99	99	2.082	9647	protein tyrosine phosphatase; non-receptor type 14
3	ENSG00000125869	LAMP5	0.0000000000	1.99	93	2.046	16097	lysosomal associated membrane prot.member 5 [HGNC:16097]
4	ENSG00000169908	TM4SF1	0.0000000001	1.96	93	2.031	11853	transmembrane 4 L six family member 1 [HGNC:11853]
5	ENSG00000113389	NPR3	0.0000000002	1.95	97	2.136	7945	natriuretic peptide receptor 3 [HGNC:7945]
6	ENSG00000186007	LEMD1	0.0000000003	1.95	85	1.937	18,725	LEM domain containing 1 [HGNC:18725]
7	ENSG00000135338	LCA5	0.0000000003	1.89	97	2.021	31,923	LCA5; lebercilin [HGNC:31923]
8	ENSG00000169826	CSGALNACT2	0.0000000008	1.91	92	1.974	24,292	chondroitin sulfate N-acetylgalactosaminyltransferase 2
9	ENSG00000059804	SLC2A3	0.0000000014	1.93	89	1.993	11,007	solute carrier family 2 member 3 [HGNC:11007]
10	ENSG00000099860	GADD45B	0.0000000018	1.92	97	2.074	4096	growth arrest and DNA damage inducible beta [HGNC:4096]
11	ENSG00000136155	SCEL	0.0000000018	1.88	87	1.928	10,573	sciellin [HGNC:10573]
12	ENSG00000100625	SIX4	0.0000000019	1.89	91	1.951	10,890	SIX homeobox 4 [HGNC:10890]
13	ENSG00000131016	AKAP12	0.0000000028	1.85	95	2.092	370	A-kinase anchoring protein 12 [HGNC:370]
14	ENSG00000158270	COLEC12	0.0000000028	1.84	92	1.941	16,016	collectin subfamily member 12 [HGNC:16016]
15	ENSG00000154553	PDLIM3	0.0000000047	1.84	91	1.985	20,767	PDZ and LIM domain 3 [HGNC:20767]
16	ENSG00000082781	ITGB5	0.0000000049	1.82	88	1.911	6160	integrin subunit beta 5 [HGNC:6160]
17	ENSG00000144366	GULP1	0.0000000050	1.81	88	1.911	18,649	engulfment adaptor PTB domain containing 1 [HGNC:18649]
18	ENSG00000171951	SCG2	0.0000000051	1.81	93	2.034	10,575	secretogranin II [HGNC:10575]
19	ENSG00000185567	AHNAK2	0.0000000066	1.80	87	1.896	20,125	AHNAK nucleoprotein 2 [HGNC:20125]
20	ENSG00000138061	CYP1B1	0.0000000075	1.84	85	1.884	2597	cytochrome P450 family 1 subfamily B member 1 [HGNC:2597]
21	ENSG00000184304	PRKD1	0.0000000451	1.74	87	1.872	9407	protein kinase D1 [HGNC:9407]
22	ENSG00000152583	SPARCL1	0.0000000471	1.74	85	1.863	11,220	SPARC like 1 [HGNC:11220]
23	ENSG00000147883	CDKN2B	0.0000000717	1.73	84	1.847	1788	cyclin dependent kinase inhibitor 2B [HGNC:1788]
24	ENSG00000213190	MLLT11	0.0000001989	1.70	84	1.813	16,997	myeloid/lymphoid or mixed-lineage leukemia; translocated to 11
25	ENSG00000135218	CD36	0.0000002751	1.69	85	1.891	1663	CD36 molecule [HGNC:1663]
1	ENSG00000133216	EPHB2	0.0000000000	0.43	100	0.426	3393	EPH receptor B2 [HGNC:3393]
2	ENSG00000169718	DUS1L	0.0000000000	0.49	98	0.481	30,086	dihydrouridine synthase 1 like [HGNC:30086]
3	ENSG00000163545	NUAK2	0.0000000001	0.51	96	0.495	29,558	NUAK family kinase 2 [HGNC:29558]
4	ENSG00000158169	FANCC	0.0000000002	0.51	95	0.498	3584	Fanconi anemia complementation group C [HGNC:3584]
5	ENSG00000277972	CISD3	0.0000000002	0.51	87	0.511	27,578	CDGSH iron sulfur domain 3 [HGNC:27578]
6	ENSG00000099800	TIMM13	0.0000000003	0.53	95	0.511	11,816	translocase of inner mitochondrial membrane 13 [HGNC:11816]
7	ENSG00000116771	AGMAT	0.0000000005	0.52	95	0.515	18,407	agmatinase [HGNC:18407]
8	ENSG00000118513	MYB	0.0000000006	0.52	93	0.508	7545	MYB proto-oncogene. Transcription factor [HGNC:7545]
9	ENSG00000016391	CHDH	0.0000000006	0.53	90	0.520	24,288	choline dehydrogenase [HGNC:24288]
10	ENSG00000137460	FHDC1	0.0000000008	0.52	96	0.505	29,363	FH2 domain containing 1 [HGNC:29363]
11	ENSG00000132846	ZBED3	0.0000000009	0.52	88	0.522	20,711	zinc finger BED-type containing 3 [HGNC:20711]
12	ENSG00000162408	NOL9	0.0000000015	0.54	92	0.527	26,265	nucleolar protein 9 [HGNC:26265]
13	ENSG00000109534	GAR1	0.0000000017	0.50	99	0.479	14,264	GAR1 ribonucleoprotein [HGNC:14264]
14	ENSG00000133477	FAM83F	0.0000000019	0.54	93	0.518	25,148	family with sequence similarity 83 member F [HGNC:25148]
15	ENSG00000100348	TXN2	0.0000000036	0.53	88	0.527	17,772	thioredoxin 2 [HGNC:17772]
16	ENSG00000108479	GALK1	0.0000000036	0.55	88	0.525	4118	galactokinase 1 [HGNC:4118]
17	ENSG00000110917	MLEC	0.0000000045	0.55	96	0.476	28,973	malectin [HGNC:28973]
18	ENSG00000114738	MAPKAPK3	0.0000000048	0.55	92	0.520	6888	mitogen-activated protein kinase-activated 3 [HGNC:6888]
19	ENSG00000137752	CASP1	0.0000000180	0.56	87	0.523	1499	caspase 1 [HGNC:1499]
20	ENSG00000131844	MCCC2	0.0000000183	0.57	93	0.516	6937	methylcrotonoyl-CoA carboxylase 2 [HGNC:6937]
21	ENSG00000178409	BEND3	0.0000000193	0.55	88	0.529	23,040	BEN domain containing 3 [HGNC:23040]
22	ENSG00000114737	CISH	0.0000000216	0.55	87	0.508	1984	cytokine inducible SH2 containing protein [HGNC:1984]
23	ENSG00000011376	LARS2	0.0000000239	0.55	91	0.528	17,095	leucyl-tRNA synthetase 2; mitochondrial [HGNC:17095]
24	ENSG00000164045	CDC25A	0.0000000481	0.57	90	0.539	1725	cell division cycle 25A [HGNC:1725]
25	ENSG00000154655	L3MBTL4	0.0000000606	0.54	90	0.506	26,677	l(3)mbt-like 4 (Drosophila) [HGNC:26677]

The first part of the table corresponds to the top-25 genes where up-regulation corresponds to shorter survival and higher risk (i.e., HR > 1); the second part of the table corresponds to the top-25 genes where UP-regulation corresponds to longer survival and lower risk (HR < 1). The genes were ranked by their KM adjusted *p* values and the Hazard Ratio values calculated for the whole dataset, i.e. for all the 1273 samples (all-dt). The stability and robustness of the gene survival markers was assessed by cross-validation, applying to each gene a resampling strategy with random selection of 80% of the samples 100 times (i.e. doing 100 iterations). For the ranking we also considered that the genes had to give a significant adjusted p-value in more than 80 iterations (N-sinf-in-100i > 80)

### External validation of prognostic markers with a CRC cohort studied using RNA-seq

The analyses done so far provided a ranked collection of genes found as robust markers of survival in CRC. The consistency of the results obtained with the internal cross-validation gives strong support to the top genes found (presented in Table 3), but we had to consider the value of using other external independent CRC cohorts to corroborate these findings. As far as we could investigate we did not find other large CRC datasets (i.e., sets with more than one thousand samples) that included global gene expression data plus survival as part of the clinical characterization of samples. Despite this limitation, we look for independent datasets and found in The Cancer Genome Atlas (TCGA, http://tcga-data.nci.nih.gov/docs/publications/coadread_2012/) a well-characterized cohort of 276 colorectal carcinomas that had been studied with several genome-scale technologies (including RNA-seq gene expression profiling) and that had survival data for 269 samples [14]. We used these data to validate the top genes found as best survival markers in our previous analysis. The results indicated a good performance in more than two thirds of the genes tested. In Additional file 4: Table S4 we present the KM *p*-values and HR of the genes that were validated from the top 10 previously found: 7 genes of the top 10 for the case of up-regulation associated with poor survival (PTPN14, LAMP5, TM4SF1, LCA5, CSGALNACT2, SLC2A3 and GADD45B) and 6 genes of the top 10 previously found for the case of up-regulation associated with good survival (EPHB2, DUS1L, NUAK2, FANCC, MYB and CHDH).

### External validation of prognostic markers using multivariate survival analysis

Up to now the search to find gene survival markers associated to the prognosis of CRC have been done using univariate analysis that look for the value and influence of each singular gene. The results presented provided multiple parameters to allow a proper statistical assessment and ranking of each gene survival markers proposed (Table 3). To provide extra support to these results we did another external validation using a second independent cohort of CRC samples from the platform SurvExpress [15]. The CRC dataset selected was called “Colon-Metabase-Uniformized” and it included 482 samples with overall survival data and genome-wide expression determined with *Affymetrix* microarrays. We performed several multivariate survival analyses (OS, overall survival) on this dataset using combinations of the top genes proposed in Table 3. As an example of these analyses we present the KM plot (Additional file 5: Figure S1) corresponding to the multivariate survival study done using the top 5 genes found up-regulated for poor survival (DCBLD2, PTPN14, LAMP5, TM4SF1 and NPR3). It can be seen that the combination of these genes provides a very good separation of two CRC populations: one group of high-risk, associated to the overexpression (or up-regulation) of the genes; and another group of low-risk, associated to the lower expression (or down-regulation) of these genes (Additional file 5: Figure S1). This analysis was repeated with several other combinations of the top up-regulated genes associated with poor survival (present in Table 3), resulting in similar results. For example, combining DCBLD2, LAMP5, TM4SF1, NPR3 and GADD45B the separation of the high and low-risk groups improved a bit: KM *p*-value = 2.21e-07 and HR = 2.23 (95% confidence interval, CI: 1.65–3.02). Another combination that provided very good separation was using genes DCBLD2, LAMP5, TM4SF1, NPR3 and AKAP12: KM p-value = 2.51e-10 and HR = 2.74 (95% CI: 2.00–3.74).

### Gene expression profiles of CRC tumor samples versus normal colorectal samples

All the integrated datasets, so far presented in this study corresponded to CRC samples, because we want to provide genes that are disease markers present in the transformed tumor cells of the intestinal epithelium, and genes that mark the progression and aggravation of this type of cancer. In addition, we can only have survival information about patients since in healthy individuals survival time cannot be related to disease and there are not disease-associated events. Despite this obvious consideration, it is interesting to explore what would be the level of expression of the genes, that we identified as survival markers, when they are analysed in normal colorectal tissue. Exploring back on the experimental series used to create our meta-dataset of 1273 CRC samples, we found in series GSE33113 and GSE39582 a collection of 25 samples that corresponded to normal colorectal tissue. We took these samples and included then with our CRC dataset using the same normalization protocol. After this integration, we could explore the expression level of the top up-regulated genes (identified as markers of poor survival), comparing the expression distribution on a set of cancer samples versus a set of normal tissue samples. In both cases the number of samples compared were 25, since this is the number of normal samples that we had. We did this comparison 20 times, random selecting each time a different subset of 25 cancer samples. The results were always very similar and the boxplots of the expression distributions for the top 10 genes are presented in Additional file 6: Figure S2. These results indicate that the gene markers, identified in our survival studies, are most of the times also up-regulated in CRC tumors with respect to normal colorectal tissue.

### Risk predictor score based in the multivariate analysis of candidate survival markers

**Fig. 4:**
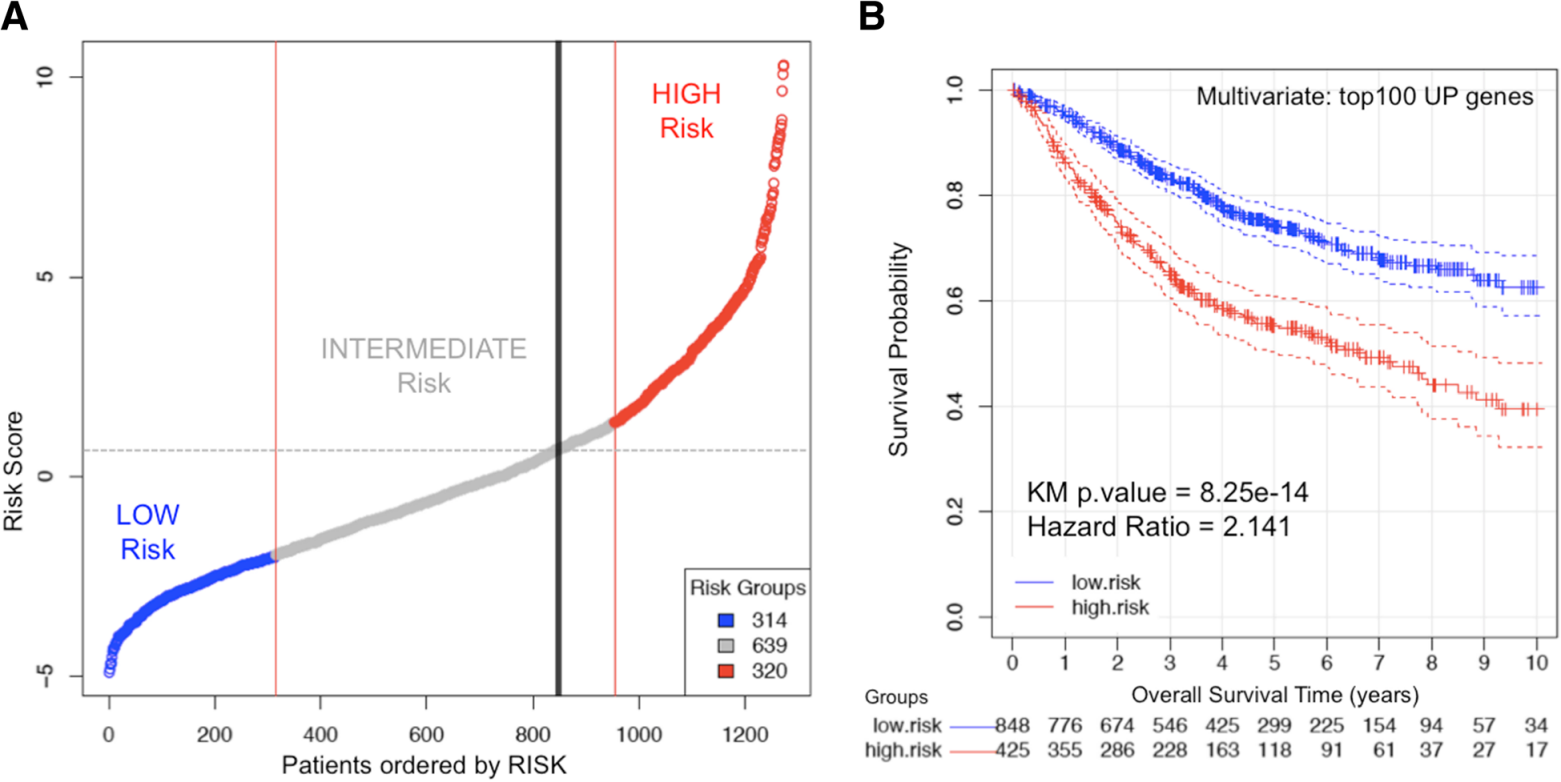
Risk prediction done for the cohort of 1273 patients of CRC based in the multivariate analysis using the top 100 genes that showed up-regulation correlated with poor prognosis (i.e. overexpressed in low survival cases). **a** Plot presenting the patients according to their risk score, from Low (blue) to High (red) risk. A recursive algorithm using 10-fold cross-validation finds the value of risk score (marked with a vertical black line) that allows the best splitting of the cohort in two groups. **b** Kaplan-Meier plot showing the separation of these two groups: a high-risk group including 425 individuals (in red) and a low-risk group including 848 individuals (in blue). The analysis has been done using a multivariate Cox proportional-hazards regression. As shown, the division is very significant (*p*-value = 8.25e-14) and allows an optimal separation of individuals according to their survival. The analysis of the *beta* factors assigned by the regression to each of the top 100 genes (i.e. to each variable within the multivariate vector) allows the identification of the genes that are the most influential factors in this risk analysis and therefore it helps in the selection of the best “gene survival markers”

Finally, to obtain a more accurate evaluation of the prognostic value of all the genes selected as best candidates (reported in Additional files 2 and 3**,** Table S2 and Table S3), we performed another analysis of the candidate markers using a regularized multivariate Cox proportional-hazards regression with L1 norm penalty [16], with the scope of building a multigenic “risk predictor”. This analysis was done on the cohort of 1273 samples of CRC patients, using for the multivariate analysis the top 100 genes that showed up-regulation correlated with poor prognosis (i.e. overexpressed in low survival cases). The results are presented in Fig. 4 that shows a graph ordering the patients according to their risk score, from low-risk (blue) to high-risk (red), including also an intermediate region (grey) (Fig. 4a). A recursive algorithm using 10-fold cross-validation was applied to find the value of risk score. The threshold (marked with a vertical black line) is obtained by maximizing the separability between the survival curves for the resulting groups. Therefore, it allows the best splitting of the cohort in two groups. A Kaplan-Meier plot showing the separation of these two groups is also presented (Fig. 4b); dividing the population into a high risk group including 425 individuals and a low risk group including 848 individuals. As shown, the division is significant (*p*-value = 8.25e-14) and allows an optimal separation of individuals according to their survival. The analysis of the beta factors assigned by the regression to each of the top 100 genes, i.e. to each variable within the multivariate vector (data included in Additional file 7: Table S5), allows the identification of the genes that were the most influential factors in this risk analysis and therefore it facilitated the selection of the best “gene survival markers”. As indicated in previous sections, the top 100 genes included in the construction of this multigenic risk predictor score were selected from the list of best markers found during the survival test with single genes.

## Discussion

CRC is a complex disease composed of biologically and clinically diverse subtypes, which can originate in different ways provoking multiple clinical scenarios [1, 2]. This complexity causes the molecular characterization of CRC to remain deficient, with a lack of clear gene markers associated to specific CRC subtypes and to the prognosis of the disease [17–19]. In fact, current molecular phenotyping of colorectal tumors is usually linked to the traditional determination of somatic mutations in well-known oncogenes such as KRAS and BRAF [20].

The recent advance of genomic and transcriptomic technologies applied to the study of clinical samples did open the way to obtain genome-wide expression profiles of multiple patient cohorts and correlate the expression of certain genes with different disease subtypes, disease stages and progression [21, 22]. This approach had been widely applied in cancer research in the last decade and is very powerful when the identification of marker genes is associated with survival time. The correlation between gene expression and survival is an excellent tool to investigate prognosis of the disease and to build risk predictors that will be applicable to individual patients.

The identification of molecular biomarkers with prognostic value in CRC has been a challenging task [23–26]. Molecular prognosis of colorectal tumor samples by transcriptional profiling started about 15 years ago (see review [24]), and in more recent years several specific gene signatures associated with CRC survival have been published [5–7, 27–31]. Despite these efforts, at present there is not a clear compendium of gene markers for CRC survival and it is quite difficult to find consistency in the literature [24]. A clear limitation comes from the fact that, in most of previous studies, the number of tumor samples used to select the genes that enter into the construction of the prognostic predictors is small (i.e., the size of the patient cohorts rarely it is greater than a few hundred individuals). For example, *ColoPrint* is a 18-gene signature for prognosis prediction of stage II and III CRC, that was identified using as training set tumor samples from 188 patients [7, 27]; a 113-gene expression signature for predicting prognosis in patients with CRC was built using 145 samples as dicovery set [28]; a 7-gene signature to predict overall survival of CRC patients was based in an initial training set of 67 samples [29]; a recurrence-associated CRC signature of 13 genes was developed using a screening set of 145 samples [30]; a 15-gene signature for prediction of CRC recurrence and prognosis was elaborated using for the gene selection a set of 55 patients [31]. In conclusion, we can say that as far as it is reflected in the current literature, the size of the initial training sets used to identify candidate gene markers for CRC survival is small and the overlap between the published gene signatures is very reduced and inconsistent. To address these critical problems, we constructed a large, well-standardized, integrated data set of 1273 tumor samples with survival information, which was used to identify genes that had a clear change in expression in the middle and late stages of CRC and were consistent markers of the disease-outcome and patient-risk.

With respect to the specific genes proposed as CRC survival markers, we want to underline that our study does not pretend to provide a fixed gene signature for prognosis and risk prediction, like the reported signatures of 7-genes, 15-genes or 113-genes [28, 29, 31]; but instead we propose a robust set of genes ranked according to their predictive power of CRC survival. In this way, an ordered list of 200 genes including the best survival markers is presented: 100 genes for which up-regulation marks “poor survival” and 100 genes for which up-regulation marks “good survival”. We think that this approach is more useful, since it allows an open selection of different number of genes for further purposes or investigations (for example, for additional tests with other CRC clinical cohorts). In fact, we used the 100 most significant genes, up-regulated with the progression of CRC, to build the risk predictor (presented in Fig. 4); and we used the top 5 or top 10 genes of this list for the external validations with different independent datasets.

Another relevant comment is that, as reminded above, we constructed the risk predictor using the genes that showed up-regulation correlated with poor prognosis. This was done because in the selection of biomarkers it is better to use the ones that provide a positive signal (i.e. “gain-of-function” factors) than the ones that provide a negative signal. Therefore, all the gene survival markers that we proposed were detectable as overexpressed in the CRC patients with high risk. The fact that they give a positive signal will also make easier their detection by standard biomolecular protocols (PCR, ELISA, immunohistochemistry, etc).

Finally, we are investigating the biological meaning of the genes found as best predictive and prognostic markers. We are focusing our efforts in the top 10 for which up-regulation marked poor survival: DCBLD2, PTPN14, LAMP5, TM4SF1, NPR3, LEMD1, LCA5, CSGALNACT2, SLC2A3, GADD45B. The analysis of the literature reveals some relevant observations. For example, the transmembrane protein DCBLD2 (ESDN), member of a family of neuropilin-like proteins, is a novel regulator of mitotic and metabolic effects of insulin, and it modulates signal transduction through regulation of the insulin receptor interaction with its adaptor proteins [32]. The importance of insulin regulation in the function of our digestive system is clear, and this adds extra value to the proposal of DCBLD2 as a CRC survival marker. Other genes within the top rank have been recently involved in cancer progression, like the case of SLC2A3 (GLUT3) a glucose transporter that mediates glucose utilization and glycogenolysis, which is induced during epithelial-mesenchymal transition and promotes tumor cell proliferation [33]. Recent publications have also proposed the role of some other genes found as prognostic markers, like the case of LAMP5 that has been included in a multigenic assay to predict recurrence for gastric cancer patients after surgery [34]. As a final example, GADD45B (growth arrest and DNA-damage-inducible 45 beta) is a gene that responds to environmental stresses, associated with cell growth control, apoptosis and DNA damage repair response. GADD45B overexpression has been recently correlated with shorter overall survival in colorectal carcinoma [35]. Moreover, a recent integrative analysis of multiple colon cancer gene-expression-based subtype classifiers reported that one of the three highest scoring genes included in several classifiers was GADD45B [36].

Despite all these positive findings that correspond to the biological value and the support of the genes identified as most significant markers of CRC survival, there are some possible limitations of the results, beginning with the general observation about the frequent heterogeneity of the colorectal tumors [1, 17]. In fact, it is clear from the anatomical pathology that CRC can affect quite different regions of the digestive tract: ascending colon, transverse colon, descending colon, sigmoid colon and rectum. The causal genes that drive tumors in these different regions may not be the same, and most CRC studies do not enter into a detailed separation of these regions [19]. The variability due to the different staging of the tumors is another factor that can bring limitations to any CRC study; but in this case we clearly indicated that our work searched for genes that were candidate prognostic markers for CRC in stages III and IV. A final reason for the limitations of the results may be an over-adjustment to the tested data sets. To avoid this kind of limitations, we built a large well-normalized data set with more than a thousand samples, performed a cross-validation analysis on that set, and also explored the validity of the gene markers in two other independent sets.

## Conclusions

In conclusion, we consider that the results presented in this work provide strong support and a solid rationale for the prognostic value of a new set of genes in CRC and for their potential to predict colorectal tumor progression and evolution towards stages III and IV. The final proposed set of gene survival markers includes an open list of one hundred up-regulated genes, with a robust statistical estimation of the value of each one. In this way the set of genes is clearly ranked, being the top in the list the ones that provide best prognostic strength and the ones that can be introduced to build smaller predictors. In fact, our results showed that a selection of the top 5 genes applied to independent external cohorts provided very good separation of CRC samples in two distinct groups of high and low risk.

## Methods

### Genome-wide expression data sets

In this study, we have analysed and integrated seven data sets of CRC samples (Table 1). All data sets are available at GEO repository, corresponding to 7 series with the following accession numbers: GSE14333, GSE17536, GSE31595, GSE33113, GSE38832, GSE39084 and GSE39582. All these series included the raw expression signal and correspond to data obtained with the microarrays expression platform: *Affymetrix GeneChip U133 Plus 2.0* for *Homo sapiens*. The phenotypic information corresponding to all these series was analysed in order to select only the samples that included information regarding: the cancer *stage* and the *Overall Survival* (OS). The samples that did not have any survival information were discarded from the study. In all cases only primary tumors samples were considered for our analysis; in this way individuals who had received preoperative chemotherapy and/or radiotherapy were also discarded.

For the external validation we used two independent datasets. A cohort of 276 colorectal carcinomas that had been studied using RNA-seq gene expression profiling, and that had survival data for 269 samples [14] (which can be found in http://tcga-data.nci.nih.gov/docs/publications/coadread_2012/). A second cohort of CRC samples from the platform SurvExpress [15]. This second dataset selected, called “Colon-Metabase-Uniformized”, included 482 CRC samples with overall survival data and genome-wide expression determined with *Affymetrix* microarrays (see the website http://bioinformatica.mty.itesm.mx:8080/Biomatec/SurvivaX.jsp).

### Expression data sets exploration and integrative normalization

Previously, to make the best use of the information obtained from the microarrays, we have considered the importance to ascertain the quality of the data. To assess the validity of generated microarray information we have performed a wide variety of quality assessment methods, both in raw and pre-processed information. In this way, several explanatory data analysis were applied for the detection of problematic arrays. We used the R function *image* to create chip images of the raw intensities to discover spatial artefacts in the samples. We have also look at the distribution of probes intensities across all arrays, using the *boxplot* method available for the Affybatch class. We also applied to the samples the Normalized Unscaled Standard Error (NUSE) algorithm. This quality assessment tool requires a previous PLM fitting procedure applied on the raw expression data. We have used the function *fitPLM* provided in the *AffyPLM* package to create the *PLMset* class object used as the input in the elaboration of the NUSE analysis. After applying the referred quality assessment methods, we discarded 79 of the initial samples collected and proceed with the remaining 1273 (Table 1).

To create a table with all the phenotypic characteristics of the patients selected which involved all samples GSM accession numbers and related clinic variables in a consistent and homogenize way, we used *getGEO* and *pData* functions from *GEOquery* package (this table is provided as Additional file 1: Table S1). We made use of regular expressions and common text manipulation R functions to solve the issue of formatting heterogenic data. Finally, we created a binary variable to label the patients and select them in a proper way during the hypothesis contrasts and statistical modeling.

### Batch effect removal

Batch effect is one of the main problems when several datasets are combined to be studied together, because different batches usually add large unwanted variability to the data. To avoid this effect we tested a combination of different pre-processing and normalization algorithms: Robust Multi-array Average (RMA) algorithm [11]; Combatting Batch effects (ComBat) algorithm [12]; Frozen Robust Multi-array Average (fRMA) algorithm [13]. For the fRMA algorithm application, we constructed the frozen parameter vector using a training dataset in where we distributed randomly selected samples proportionally to each labelled group to obtain a balanced sample from the 7 batches of microarrays.

Another important issue addressed was the fact that the *Affymetrix* probe-sets included in the expression microarrays many times do not correspond to singular genes and some probes inserted in the defined probe-sets are ambiguous or inaccurate [10]. *Affymetrix* GeneChip is a popular and usefull platform for gene expression profiling, but the use of its probes and probe-sets mapping has multiple inconveniences. In fact, the probe-sets for the *Affymetrix* Human Genome U133 Plus 2.0 Array are based on UniGene database (Build 133, April 20, 2001) and considering how rapidly human genome has evolved many probes on the array are not correctly assigned. To avoid this problem, we used the updated probe alignment and gene mapping that is provided by the Chip Definition File (CDF): *hgu133plus2hsensgcdf* (downloaded from http://brainarray.mbni.med.umich.edu/).

### Batch effect removal evaluation

We performed unsupervised hierarchical clustering to observe unlikely clustering based on batches in those expression value matrixes where batch effects remained after pre-processing. We used a 30-random sampling per batch, identifying each batch by a different color (Fig. 1). The batch effect was also investigated using principal components analysis (PCA) (Fig. 2). A linear regression of average gene expression on array batch per pre-processing method was the final approach fulfilled to assure removal (Table 2).

### Differential expression analysis

For the identification of gene whose altered expression achieved statistical significance we used the R algorithm Linear Models for Microarrays (LIMMA package). We applied LIMMA to the expression data matrix fixing an adjusted *p*-value threshold of FDR ≤ 0.01 to select significant genes. The comparison was done separating the samples according to their clinical and pathological stage (comparing CRC stages I and II versus III and IV). In this way we found a set of 2707 candidates genes, corresponding to 2524 protein-coding genes that were tested in the survival analysis (the rest were non-coding genes). In this work we focus only on the genes that encode proteins because we wanted to find CRC survival markers that later can be tested at protein level using, for example, immunohistochemistry (IHC) analysis.

### Survival analysis

Our intention in this research was to identify genes whose relative expression level affect survival and prognosis in CRC, once we had made a preselection in its behavior through stage evolution of 2524 protein-coding genes.

The first step for the survival analysis was to define for each gene two separated distributions of high and low expression along the sample dataset investigated. This separation based in expression level determined the explanatory variable. We used the *Surdiff* function in the *Survival* package to address the issue. By sorting all the samples in ascending order, we performed *Surdiff* hypothesis testing, splitting the group of samples for each gene and every sample between quantile 25% and 75% to obtain its Chi-square associated p-value. Then we selected minimum p-value to perform final group assignation of high and low expression. Once we had the two groups clearly defined, we used the *Coxph* model to obtain each associated p-value and hazard ratio (HR) from every candidate gene. In this way, the survival analysis along the two groups also allowed estimating hazard ratios (HR) or, what is the same, tried to measure how the expression, in terms of high and low relative expression for each candidate gene, altered the hazard function. Finally, for computing the time to event, the response variable in the models was the *Overall Survival* (OS) time. All the data sets that we integrated in our analyses had OS information. In some cases for some individuals, *Disease Specific Survival* (DSS) times or *Relapse Free Survival* (RFS) times were also provided with the original data, but we did not considered these time-events since we wanted to focus on OS to achieve a homogeneous analysis.

## Additional files

Additional file 1: **Table S1.** Phenotypic and clinical information about the collection of 1273 colorectal cancer samples that has been integrated in this work. The table includes the IDs of the samples in GEO and all the available data about age, gender, survival time, location of the tumor, degree and TNM staging, presence of mutation in some cancer genes (TP53, KRAS, BRAF), etc. When information was not available for a given sample the table includes NA (not available values). (XLSX 272 kb)

Additional file 2: **Table S2.** Top-100 best survival marker genes for colorectal cancer (CRC) that are up-regulated when survival is poor and the risk is higher (i.e., HR > 1). This table is an expension of the data in Table 3. The genes were ranked by their KM adjusted *p*-values and the HR values calculated for the whole dataset (i.e. for all the 1273 samples = all-dt). The stability of each survival marker gene was assessed by cross-validation (100 iterations). The table also includes the number of times that a survival marker was significant in the iterations (N-sinf-in-100i). (XLSX 73 kb)

Additional file 3: **Table S3.** Top-100 best survival marker genes for colorectal cancer (CRC) that are down-regulated when survival is poor and the risk is higher (i.e., HR < 1). This table is an expension of the data in Table 3. The genes were ranked by their KM adjusted p-values and the HR values calculated for the whole dataset (i.e. 1273 samples = all-dt). The stability of each survival marker gene was assessed by cross-validation (100 iterations). The table also includes the number of times that a survival marker was significant in the iterations (N-sinf-in-100i). (XLSX 70 kb)

Additional file 4: **Table S4.** Validation of the survival data done in an independent set of samples taken from The Cancer Genome Atlas (TCGA), that included 269 colorectal carcinomas with survival information and RNA-seq global expression profiling. The table includes the KM p-values and HR of the genes that were validated from the top-10 survival marker genes previously found presented in Table 3. Of the top-10 for the case of up-regulation associated with poor survival, 7 were validated (PTPN14, LAMP5, TM4SF1, LCA5, CSGALNACT2, SLC2A3 and GADD45B). Of the top-10 found for down-regulation associated with poor survival, 6 genes were validated (EPHB2, DUS1L, NUAK2, FANCC, MYB and CHDH). (XLSX 51 kb)

Additional file 5: **Figure S1.** Survival multivariate analysis of an independent set of 482 samples of CRC patients carried out considering the expression profiles of 5 genes: DCBLD2, PTPN14, LAMP5, TM4SF1 and NPR3. **(A)** Kaplan-Meier plot presenting the patients divided in two groups according their risk score: High risk (red) and Low risk (green). **(B)** Box plots showing the distributions of global expression corresponding to these 5 genes. For each gene, the dataset of 482 samples was divided in the two groups of patients indentified as High risk (red) and Low risk (green). (PDF 356 kb)

Additional file 6: **Figure S2.** Comparison of the distributions of the expression signal corresponding to ten genes in 25 samples from normal colorectal epithelium (green boxplots) versus 25 samples from CRC (red boxplots). The genes selected for this analysis were the top-10 best survival marker genes found up-regulated for poor prognosis (i.e. markers up-regulated when there is low CRC survival): DCBLD2, PTPN14, LAMP5, TM4SF1, NPR3, LEMD1, LCA5, CSGALNACT2, SLC2A3 and GADD45B. The tumor samples were not selected by stage (i.e. they were selected from any CRC stage: I, II, III or IV) and this comparison was done 20 times with different subsets of 25 CRC samples to check the stability of the signal. The plots of all the other comparisons were very similar to the plot here presented. (PDF 46 kb)

Additional file 7: **Table S5.** Beta factors assigned by regression analysis to each of the top-100 survival marker genes. These genes are taken as variables within the multivariate Kaplan-Meier survival analysis included in Fig. 4b. The factors allowed the identification of the genes that were the most influential variables in this risk analysis (i.e. the higher the better) and therefore facilitate an additional evaluation of each survival marker gene. (XLSX 62 kb)

## Abbreviations

CDF: Chip definition file
CRC: Colorectal cancer
DSS: Disease specific survival
GEO: Gene expression omnibus database
GSE: GEO Series (set of sample files that together form a single experiment)
HR: Hazard ratio
IHC: Immunohistochemistry
KM: Kaplan-Meier hazard ratio
LIMMA: Linear models for microarray data analysis
OS: Overall survival
PCA: Principal component analysis
RFS: Relapse free survival
RMA: Robust multi-array average algorithm
TCGA: The cancer genome atlas
