# Supplementary figures and images for: Survival marker genes of colorectal cancer derived from consistent transcriptomic profiling
Source: BMC Genomics. 2018 Dec 11;19(Suppl 8):857. doi: 10.1186/s12864-018-5193-9 (PMC6288855; doi:10.1186/s12864-018-5193-9)

Figure S1

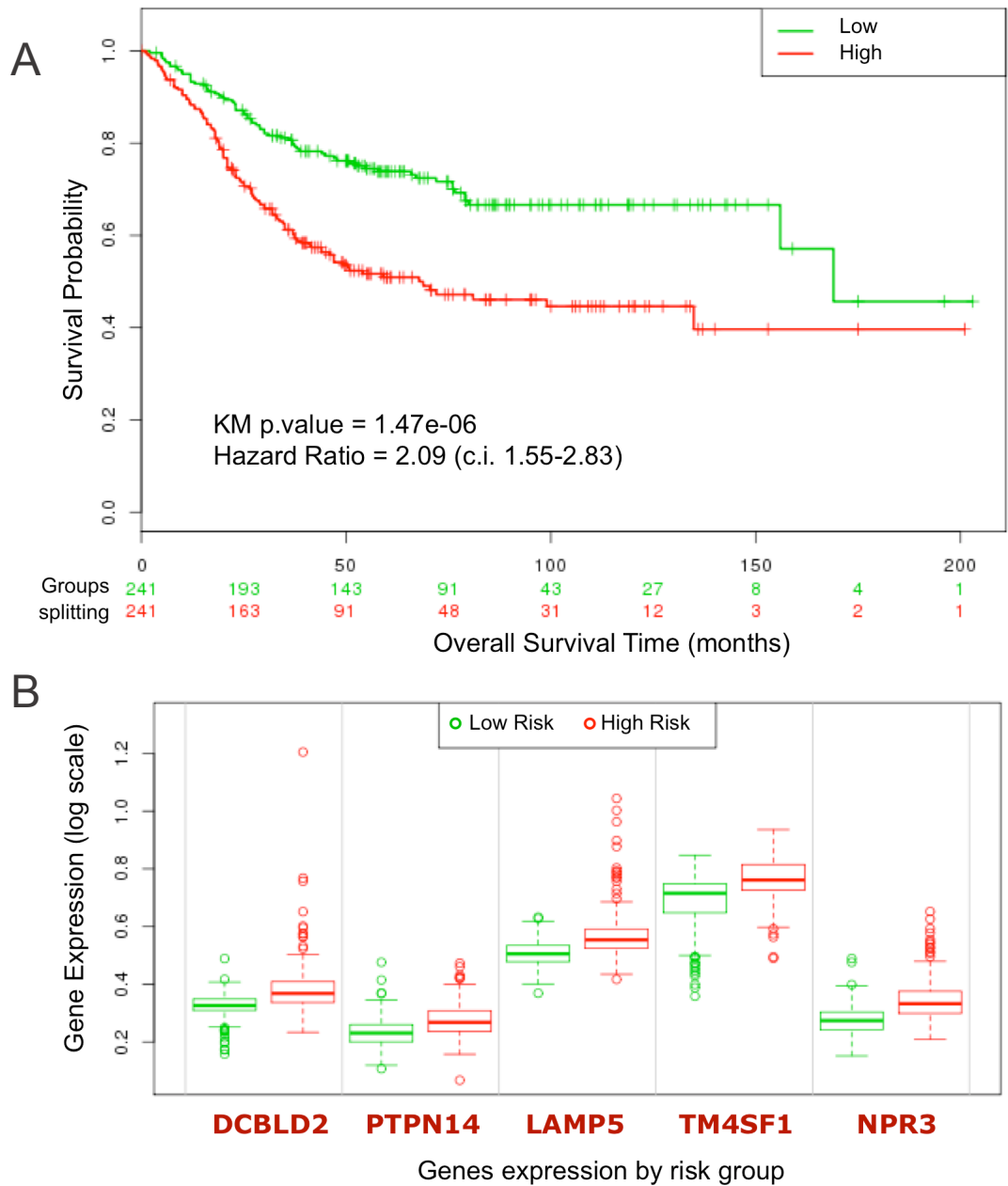

Supplement: Supplementary file 5 — Figure S1. Survival multivariate analysis of an independent set of 482 samples of CRC patients carried out considering the expression profiles of 5 genes: DCBLD2, PTPN14, LAMP5, TM4SF1 and NPR3. (A) Kaplan-Meier plot presenting the patients divided in two groups according their risk score: High risk (red) and Low risk (green). (B) Box plots showing the distributions of global expression corresponding to these 5 genes. For each gene, the dataset of 482 samples was divided in the two groups of patients indentified as High risk (red) and Low risk (green). (PDF 356 kb) [file 12864_2018_5193_MOESM5_ESM.pdf]

Figure S2

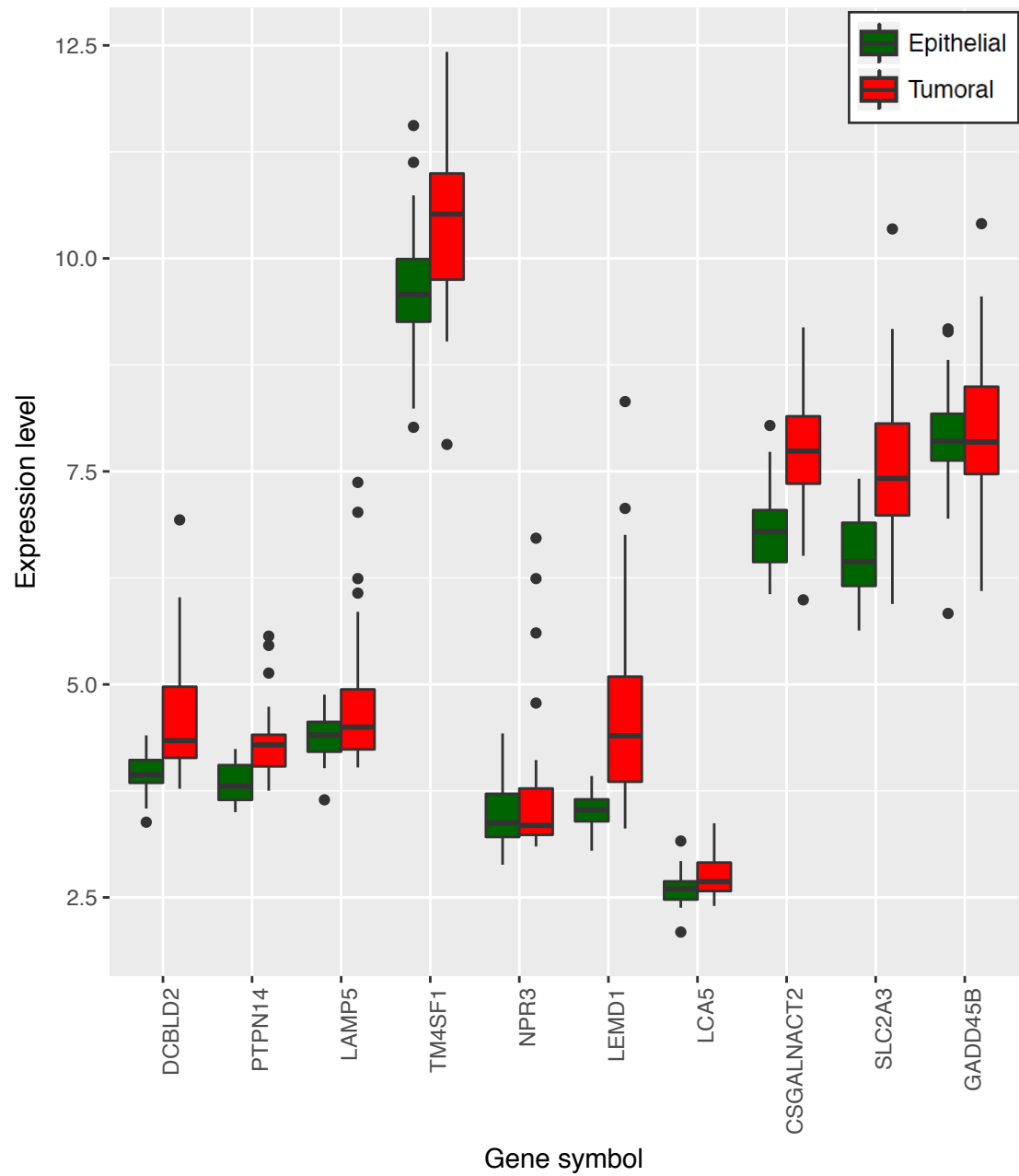

Supplement: Supplementary file 6 — Figure S2. Comparison of the distributions of the expression signal corresponding to ten genes in 25 samples from normal colorectal epithelium (green boxplots) versus 25 samples from CRC (red boxplots). The genes selected for this analysis were the top-10 best survival marker genes found up-regulated for poor prognosis (i.e. markers up-regulated when there is low CRC survival): DCBLD2, PTPN14, LAMP5, TM4SF1, NPR3, LEMD1, LCA5, CSGALNACT2, SLC2A3 and GADD45B. The tumor samples were not selected by stage (i.e. they were selected from any CRC stage: I, II, III or IV) and this comparison was done 20 times with different subsets of 25 CRC samples to check the stability of the signal. The plots of all the other comparisons were very similar to the plot here presented. (PDF 46 kb) [file 12864_2018_5193_MOESM6_ESM.pdf]
